# Effects of the small molecule SIRT1 activator, SRT2104 on arterial stiffness in otherwise healthy cigarette smokers and subjects with type 2 diabetes mellitus

**DOI:** 10.1136/openhrt-2016-000402

**Published:** 2016-05-17

**Authors:** Sowmya Venkatasubramanian, Radzi M Noh, Shruti Daga, Jeremy P Langrish, Nicholas L Mills, Brian R Waterhouse, Ethan Hoffmann, Eric W Jacobson, Ninian N Lang, Brian M Frier, David E Newby

**Affiliations:** 1 Centre for Cardiovascular Science, University of Edinburgh, Edinburgh, UK; 2 Department of Diabetes, Royal Infirmary, Edinburgh, UK; 3 GlaxoSmithKline, London, UK; 4 Sirtris, a GSK Company, Cambridge, Massachusetts, USA; 5 GlaxoSmithKline, Philadelphia, Pennsylvania, USA

**Keywords:** SIRT1, pulse wave, arterial stiffness, cigarette smokers, diabetes mellitus

## Abstract

**Objective:**

Arterial stiffness increases with age, and is associated with adverse cardiovascular outcome including increased mortality. The effect of the oral small molecule SIRT1 activator, SRT2104, on arterial stiffness was examined in otherwise healthy cigarette smokers and participants with type 2 diabetes mellitus.

**Methods:**

24 otherwise healthy cigarette smokers and 15 people with stable type 2 diabetes were randomised in a double-blind placebo-controlled crossover trial and received 28 days of oral SRT2104 (2.0 g/day) or matched placebo. Blood pressure was measured using non-invasive oscillatory sphygmomanometry. Pulse wave analysis and velocity were measured using applanation tonometry at baseline and the end of each treatment period. Owing to the small sample size and similar trends for both groups, data for the two groups were pooled (post hoc analysis).

**Results:**

Compared to placebo, treatment with SRT2104 was associated with a significant reduction in augmentation pressure (p=0.0273) and a trend towards improvement in the augmentation index and corrected augmentation index (p>0.05 for both). However, no changes were observed in pulse wave velocity and time to wave reflection (p>0.05). Systolic and diastolic blood pressures remained unchanged throughout the study. Treatment by cohort interaction was not significant for any of the pulse wave parameters, suggesting that the response to SRT2104 in otherwise healthy smokers and people with diabetes was consistent.

**Conclusions:**

SRT2104 may improve measures of arterial stiffness in otherwise healthy cigarette smokers and in participants with type 2 diabetes. Definitive conclusions are not possible given the small sample size and exploratory nature of this analysis.

**Trial registration number:**

NCT01031108.

**Key questions**

**What is already known about this subject?**

- Among the seven known sirtuins, SIRT1 has been identified as the most critical modulator of vascular function. Animal and laboratory studies have amply demonstrated its prominent role in the regulation of vascular homeostasis and diseases. However, little is known about their direct vascular effects in man.

**What does this study add?**

- The present study has provided evidence that suggests treatment with the oral SIRT1 activator, SRT2104, may lead to an improvement in measures of arterial compliance in otherwise healthy cigarette smokers and people with type 2 diabetes. The exact mechanism of this improved arterial compliance and the effects of prolonged treatment with SRT2104 on vascular health remain to be elucidated.

**How might this impact on clinical practice?**

- Given that aortic stiffness and endothelial function are key factors in predicting cardiovascular outcomes, identification of novel pharmacological means of improving these predictive parameters is important and highly relevant in populations with known cardiovascular risk factors.

## Introduction

The enzyme sirtuin (silent mating-type information regulation 2 homologue) 1 (SIRT1) belongs to the sirtuin family of nicotinamide adenine dinucleotide-dependent histone deacetylases and is highly expressed in the vascular endothelium.^1^ In addition to other characteristics, its activation is associated with improved endothelial function^2^ and inhibition of atherogenesis.^3^ Particular interest has been focused on the potential of therapeutic SIRT1 activators to act as anti-ageing agents.

Arterial stiffness rises with age and is recognised to be an independent predictor of cardiovascular risk.^4^ In particular, elevations in pulse pressure and aortic stiffness are associated with increased risk of coronary events and overall mortality.^5^ Indeed, central aortic stiffness is associated with the presence of coronary atherosclerosis and ischaemic heart disease.^6^

Cigarette smoking and diabetes mellitus are significant risk factors for the development of cardiovascular disease. A wealth of data has established a strong correlation between diabetes and cigarette smoke exposure with increased aortic stiffness, endothelial dysfunction and cardiovascular risk.^7–10^ New pharmacological strategies that improve arterial compliance would therefore be highly relevant to these groups at increased cardiovascular risk.

The aims of the present study were to assess the effect of the oral SIRT1 activator, SRT2104, on measures of arterial compliance in otherwise healthy cigarette smokers and patients with type 2 diabetes. It was hypothesised that SIRT1 activation in these ‘at risk’ groups could lead to an improvement in arterial compliance and therefore reduce their cardiovascular risk.

## Methods

The study was approved by the Berkshire Research Ethics Committee, received Clinical Trial Authorisation from the Medicines and Healthcare products Regulatory Agency (MHRA, UK), and was conducted at the MHRA phase I accredited Wellcome Trust Clinical Research Facility at the Royal Infirmary of Edinburgh, UK between June 2010 and September 2011 (EudraCT #: 2009-016765-28; Clinical trials identifier: NCT01031108). Written informed consent was obtained from each volunteer and the study was carried out in accordance with the declaration of Helsinki.

### Study participants

Twenty-four otherwise healthy cigarette smokers and 15 participants with stable type 2 diabetes, aged between 18 and 70 years, were eligible for the study. Healthy cigarette smokers were required to have smoked ≥10 cigarettes daily for at least 1 year. Participants with type 2 diabetes were non-smokers and were selected on the basis of having a diagnosis of type 2 diabetes mellitus for at least 6 months prior to inclusion in the study, with no change in medications having been made for at least the preceding 3 months, a fasting blood glucose ≤13.9 mmol/L (250 mg/dL) and diabetes control and complications trial-aligned HbA1c<9% (75 mmol/mol) on screening. Exclusion criteria included the presence of significant comorbidities, chronic illness, renal or liver impairment, history of gastrointestinal diseases or previous surgical procedures that would influence drug absorption, history of alcoholism, history of neoplastic disease within the last 5 years, a positive urinary test for recreational drugs, pregnancy and participation in other clinical trials or blood donation within the last 3 months. Patients with type 2 diabetes mellitus on ACE inhibitors, antiplatelet or anticoagulant therapies were excluded from the study. Tests for pregnancy (serum human chorionic gonadotropin (HCG) concentrations at screening and urinary HCG concentrations at study visits) were conducted on all female participants of childbearing potential.

### Study design

This was a prospective double-blind randomised placebo-controlled cross-over study. Participants were randomised to receive 2.0 g daily of oral SRT2104 or matched placebo (Sirtris, a GSK company, Massachusetts, USA) for a 28-day period, followed by cross-over to the alternate study arm for a further 28 days, giving a total dosing duration of 56 days. An end of study visit was conducted at day 70 with a telephone call follow-up on day 86. Measures of arterial stiffness were undertaken prior to and at the end of each 28-day trial period. Figure 1 outlines participant enrolment, intervention allocation, follow-up and data analysis for both groups.

**Figure 1:**
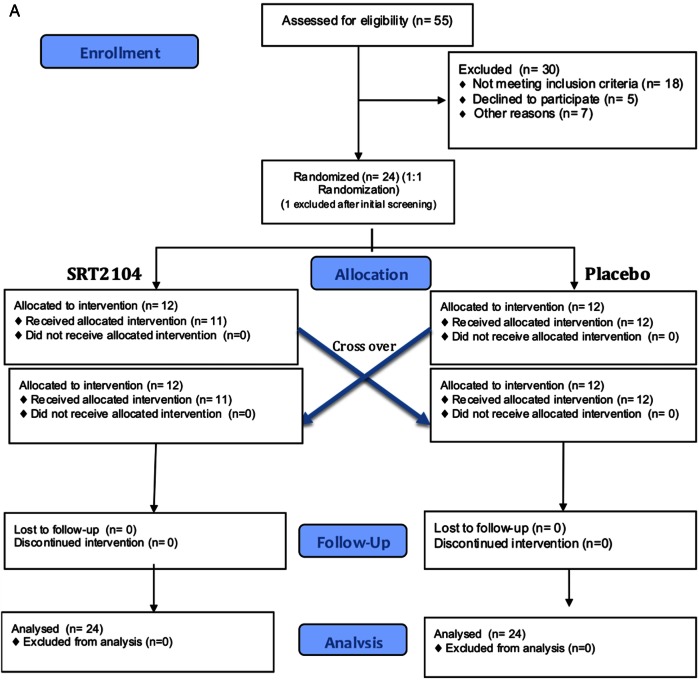
Consolidated Standards of Reporting Trials flow diagram representing participant enrolment, intervention allocation, follow-up and data analysis. (A) Recruitment of otherwise healthy cigarette smokers; (B) participants with type 2 diabetes mellitus.

**Figure 1:**
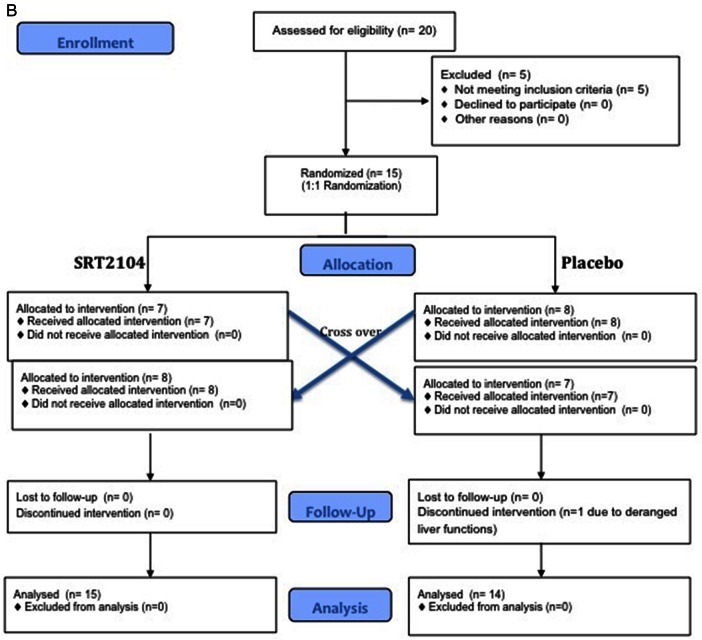
Continued

All studies were performed in a quiet temperature controlled (22–25°C) room. Participants were fasted and asked to refrain from smoking for 10 h, and abstain from caffeine and alcohol for 24 h prior to assessment. Participants remained supine for at least 30 min before any recordings were started. Systolic and diastolic blood pressures were recorded using a non-invasive oscillatory sphygmomanometer (Omron705 IT, Omron Healthcare Europe, the Netherlands).

Pulse wave analysis of the radial artery was performed at the wrist using micromanometer applanation tonometry (Millar Instruments, Texas, USA) and the SphygmoCor system (AtCor Medical, Sydney, Australia) in accordance with the manufacturer's recommendations. Briefly, pulse wave analysis derives an aortic pulse pressure waveform from the radial artery wave via a mathematical transfer function. The arterial pressure waveform is a composite of the forward pressure wave created by ventricular contraction and a reflected wave generated by peripheral vascular resistance.^11^ The augmentation pressure is the pressure difference between the second and first systolic peaks. The augmentation index, augmentation pressure as a percentage of the pulse pressure, is a measure of systemic arterial stiffness and wave reflection. Corrected augmentation index represents the augmentation index corrected for heart rate.^12^ The time to wave reflection declines with increasing arterial stiffness, and provides a surrogate measure of aortic pulse wave velocity.^13^ At least three independent waveform analyses were obtained from each participant, with measurements only accepted on meeting SphygmoCor quality control criteria. Pulse wave velocity was calculated by measuring the time for the pulse wave to travel between the carotid and femoral arteries. The operator performing the analysis was kept constant for each participant throughout the study.

### Blood sampling

Venous blood samples were collected at fortnightly intervals to measure haematological and biochemical analytes including full blood count, coagulation profile, liver and renal function, creatine kinase, lactate dehydrogenase and lipid profile. Analyses were conducted by the regional clinical haematology and biochemistry reference laboratories using an automated haematology analyser (XE2100, Sysmex Corporation (Japan) and ACL TOP, Instrumentation Laboratory), an automated chemistry analyser using colorimetric, kinetic and enzymatic ultraviolet and colour assays (AU2700/AU640 analysers, Beckman and Coulter), ion selective electrodes (sodium, potassium and chloride assays) and two point and multiple point rate assays (Ortho Clinical Vitros 250 analyser, USA).

### Data analysis and statistics

Data were analysed, where appropriate, using repeated measure analysis of covariance on the change from baseline for all parameters. Initially, analyses were conducted separately on cohorts. As a result of the small sample size and similar trends for the two cohorts, these data were pooled post hoc. Treatment differences were investigated in a model adjusting for baseline, period, treatment by period and treatment by cohort using SAS for UNIX (V.9.1.3 or higher) (SAS Institute, Cary, North Carolina, USA). Unless stated otherwise, values are expressed as mean±SD. Tests for treatment effect were two-sided with a significance level of 0.05.

## Results

### Baseline characteristics

Participants in the study had a mean age of 45±15 years and were predominantly male (68%). Participants in the type 2 diabetes cohort were older (mean age 58±8 years) when compared with the participants in the otherwise healthy smokers group (mean age 38±13 years). All participants were normotensive with comparable systolic and diastolic blood pressures at baseline (table 1). No clinically significant changes in haematological or biochemical analytes occurred throughout the study. Biochemical measures of renal function (serum urea, creatinine and electrolytes) were within normal limits at baseline and remained unchanged with placebo and treatment with SRT2104 in both subgroups (table 2).

**Table 1:** Baseline characteristics of participants who were otherwise healthy cigarette smokers or who had type 2 diabetes mellitus

	Otherwise healthy cigarette smokers (n=24)	People with type 2 diabetes (n=15)
Mean age (years)	38±13	58±8
Sex		
Male	14 (58)	13 (87)
Female	10 (42)	2 (13)
Baseline blood pressure (mm Hg)		
Systolic	129±6	133±7
Diastolic	77±2	80±3
Heart rate (bpm)	68±1	77±5
Body mass index (kg/m^2^)	25±4	30±4
Smoking history		
Number of cigarettes/day	17±6	–
Number of pack years	16	–
Urinary cotinine concentration (ng/mL)	1352±950	–
Glycaemic profile		
Baseline blood glucose (mg/dL)	85±0	144±2
HbA1c (%)	–	7.4±0.8
Concomitant medications		
Antiplatelet agents	–	5 (33%)
Antihypertensive agents	–	3 (20%)
ARB	–	2 (13%)
Diuretics	–	10 (67%)
Lipid lowering agents	–	13 (86%)
Hypolycaemic agents	–	4 (27%)
Biguanide	–	2 (13%)
Sulfonamide	–	1 (6%)
Thiazolidine	–	2 (13%)
Insulin		
Others		

Values expressed as mean±SD.

ARB, angiotensin receptor blocker; HbA1c, haemoglobin A1c.

**Table 2:** Changes in biochemical measures of renal function in otherwise healthy cigarette smokers and participants with type 2 diabetes mellitus administered placebo and SRT2104

	Otherwise healthy cigarette smokers (n=24)				Participants with type 2 diabetes (n=15)			
	Treatment period 1: placebo (n=13)		Treatment period 1: SRT2104 (n=11)		Treatment period 1: placebo (n=8)		Treatment period 1: SRT2104 (n=7)	
	Day 1	Day 28	Day 1	Day 28	Day 1	Day 28	Day 1	Day 28
Serum urea (mg/dL)	17±3	14±3	11±3	14±3	17±3	17±3	17±6	14±3
Serum creatinine (mg/dL)	0.8±0.1	0.8±0.1	0.7±0.1	0.8±0.1	0.8±0.1	0.8±0.1	0.9±0.1	0.9±0.1
Electrolytes								
Sodium (mmol/L)	140±2	140±2	140±2	140±1	139±1	139±3	138±2	139±3
Potassium (mmol/L)	4.0±0.4	4.0±0.4	4.0±0.4	4.0±0.2	4.0±0.3	4.0±0.3	4.0±0.2	4.0±0.4
Chloride (mmol/L)	109±3	107±3	108±3	107±2	104±4	104±3	103±2	104±2

Values expressed as mean±SD.

### Blood pressure

Resting systolic and diastolic blood pressures remained unchanged throughout the study with no significant differences between treatment and placebo treatment periods.

### Pulse wave analysis and velocity

**Figure 2:**
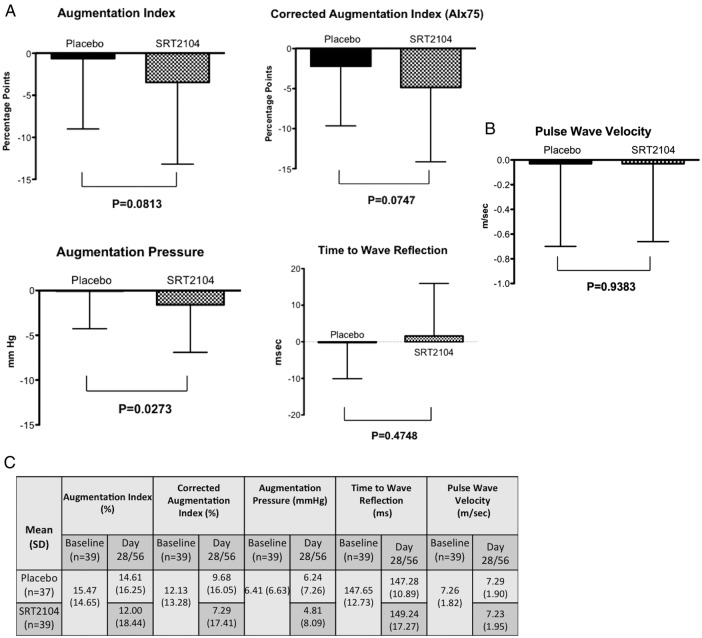
Effect of treatment with SRT2104 on measures of arterial compliance in otherwise healthy cigarette smokers and participants with type 2 diabetes mellitus–change from baseline. (A) Pulse wave analysis—augmentation index, corrected augmentation index, augmentation pressure and time to wave reflection. (B) pulse wave velocity. Solid column: placebo; checked column: SRT2104. (C) baseline parameters of measures of arterial compliance-combined data.

In a combined analysis of otherwise healthy cigarette smokers and participants with type 2 diabetes, a reduction in the augmentation pressure was observed in participants receiving SRT2104 compared with placebo (mean change from baseline: SRT2104−1.60 (5.304) vs placebo−0.06 (4.205); p=0.0273) and a trend towards improvement in the augmentation index (mean change from baseline in AIx: placebo–0.64 (8.361) vs SRT2104−3.47 (9.728); p=0.0813) and the corrected augmentation index (mean change from baseline AIx75: placebo−2.2−(7.453) vs SRT2104−4.84 (9.299); p=0.0747) (figure 2A). Pulse wave velocity and time to wave reflection remained unchanged between placebo and treatment arms (p>0.05 for both parameters; figure 2B). The effects of SRT2104 administration on measures of arterial compliance were consistent across the two cohorts. For example, in the SRT2104 arm, mean augmentation index at 75 bpm was reduced for healthy smokers and participants with type 2 diabetes (−4.97 vs −4.63, respectively). Measures of arterial compliance and stiffness for the individual cohorts have been presented in the online supplementary table S1. A statistical interaction between cohort and treatment was not observed (p>0.05 for all variables tested).

10.1136/openhrt-2016-000402.supp1 Supplementary table 1 Parameters of arterial compliance for otherwise healthy cigarette smokers and subjects with type 2 diabetes mellitus

### Tolerability and safety

Participants in both study groups (healthy cigarette smokers and patients with type 2 diabetes) tolerated the study medication well. There were no meaningful differences in the number of adverse events between active treatment and placebo. All reported adverse events were mild in intensity and resolved without any intervention or sequelae (table 3). Headaches occurred with nearly equal frequency in the treatment (SRT2104) group in both cohorts. Participants with type 2 diabetes appeared to have more frequent gastrointestinal disturbances, such as diarrhoea and nausea in comparison with healthy smokers. Elevated liver enzymes (alanine transaminase) resulted in withdrawal of one participant in the placebo period (day 36) of the diabetes group. There was only one reported serious adverse event in the study (SRT2104 arm of healthy cigarette smokers) of traumatic facial bone fracture that was considered unrelated to SRT2104.

**Table 3:** Summary of treatment emergent adverse events occurring in two or more participants in OHS and participants with T2DM

		Number of events			
		OHS		T2DM	
System organ class	Adverse event	Placebo (n=24)	SRT2104 (n=24)	Placebo (n=14)	SRT2104 n=15)
Any event		18	18	11	14
Nervous system disorders	Any event	6 (25%)	11 (46%)	1 (7%)	7 (47%)
	Headache	4	6	1	5
	Paraesthesia	0	2	0	1
	Hypoaesthesia	1	2	0	0
	Presyncope	1	1	0	0
Respiratory, thoracic and mediastinal disorders	Any event	1 (4%)	3 (13%)	0	3 (20%)
	Oropharyngeal pain	1	2	0	0
	Rhinorrhoea	0	1	0	1
Gastrointestinal disorders	Any event	3 (13%)	1 (4%)	4 (29%)	8 (53%)
	Diarrhoea	0	0	2	4
	Nausea	0	0	1	2
	Abdominal pain upper	1	0	0	1
	Dyspepsia	0	0	0	2
Reproductive system and breast disorders	Any event	3 (13%)	1 (4%)	0	0
	Dysmenorrhea	3	1	0	0
Musculoskeletal and connective tissue disorders	Any event	4 (17%)	1 (4%)	0	2 (13%)
	Back pain	2	0	0	0
Investigational	Any event	2 (8%)	1 (4%)	1 (7%)	1 (7)
	Blood bilirubin increased	1	1	0	0
	Alanine amino transferase increased	0	0	1	0
	Abnormal liver function test	0	0	0	1
General disorders and administration site conditions	Any event	4 (17%)	4 (17%)	2 (14%)	3 (20%)
	Influenza like illness	1	0	0	1
	Fatigue	1	0	1	1
Infections and infestations	Any event	3 (13%)	5 (21%)	3 (21%)	1 (7%)
	Nasopharyngitis	0	1	3	0
	Rhinitis	2	2	0	0
	Upper respiratory tract infection	1	0	0	1
Injury, poisoning and procedural complications	Any event	4 (17%)	2 (8%)	3 (21%)	1 (7%)
	Contusion	1	0	1	0
	Excoriation	1	1	0	0
Skin and subcutaneous tissue	Any event	1 (4%)	0	1 (7%)	3 (20%)
	Pruritus	0	0	0	2
Metabolism and nutrition disorders	Any event	0	1 (4%)	2 (14%)	2 (13%)
	Hypoglycaemia	0	0	1	2
Vascular disorders	Any event	0	1 (4%)	0	2 (13%)
	Flushing	0	0	0	2

OHS, otherwise healthy cigarette smokers; T2DM, type 2 diabetes mellitus.

## Discussion

This randomised double-blinded cross-over study demonstrated for the first time that the oral SIRT1 activator, SRT2104, may improve arterial compliance in otherwise healthy cigarette smokers and in people with type 2 diabetes, without affecting resting measures of blood pressure.

The assessment of arterial stiffness is increasingly being used in clinical practice as an independent measure of cardiovascular risk, including those in high-risk groups.^14^ Ageing is associated with an increase in the stiffness of large elastic arteries induced by structural alterations in the vascular media such as an increase in collagen and a decrease in elastin content.^15^ This process of biological ageing is accelerated in the presence of conditions such as diabetes mellitus and hypertension. Semba *et al*^16^ and Hofmann *et al*^17^ have demonstrated an association between the presence of advanced glycation end products and increased arterial stiffness. Indeed, vascular change induced by cigarette smoke is considered to be a model of accelerated vascular ageing. The relationship between tobacco exposure,^9^ ^10^ ^18^ diabetes^7^ ^8^ ^19^ ^20^ and increased arterial stiffness is well established.

Calorie restriction can attenuate age-related arterial stiffness in animal models through reduced oxidative stress and altered endothelial nitric oxide bioavailability.^15^ Indeed, calorie restriction can extend lifespan in lower organisms and mammals, and improves several metabolic and inflammatory parameters.^21–26^ SIRT1 has been implicated as an important mediator of lifespan extension mediated by calorie restriction.^27–29^ The current hypothesis, therefore, was that activation of SIRT1 may inhibit this process of vascular ageing and be associated with improvements in arterial stiffness.

No studies have examined the direct effect of SIRT1 activation on measures of arterial compliance. Botden *et al*^30^ were unable to demonstrate an improvement in augmentation index or central or peripheral blood pressure following treatment with red wine polyphenols. In the present study, a 28-day period of treatment with the oral SIRT1 activator SRT2104 was associated with a reduction in augmentation pressure and trends towards improvement in augmentation index and corrected augmentation index. Augmentation pressure and index are measures of arterial compliance and wave reflection from small to medium sized arteries. As such, they can be influenced by endothelial function and a number of other dynamic and functional factors, such as heart rate and peripheral circulatory tone.^7^ ^14^ Preclinical studies have demonstrated improved vascular function with SIRT1 activation,^2^ ^31–33^ and this may explain our observations of improvement in dynamic measures of arterial stiffness following short-term administration of SRT2104.

Pulse wave velocity is a more direct measure of arterial stiffness that is determined by the structural and physical composition of the arterial wall.^8^ Changes in pulse wave velocity are therefore more gradual and less dependent on the function of small to medium sized arteries. In the present study, a change in pulse wave velocity was not observed with SRT2104 administration. This is perhaps not surprising given the short-time period of exposure to SRT2104 (28 days) and the brief period of observation. An improvement in pulse wave velocity might be anticipated with a longer period of treatment with SRT2104, to allow more favourable structural changes in the larger arterial tree.

## Study limitations

Some limitations of this trial should be considered. Although favourable trends in parameters of arterial compliance were observed, some did not achieve statistical significance. This may partly be attributed to the trial being designed specifically to examine the acute effects of treatment with SRT2104. A longer period of treatment may be required for benefits to emerge on variables such as pulse wave velocity that involve structural changes in the arterial wall. Moreover, the sample sizes of the two groups examined were small. Two disparate populations were studied in this trial, in whom the mechanisms of vascular dysfunction may be very different. However, the direction of beneficial effects on treatment with SRT2104 was similar between the two groups, providing reassurance of a consistency of effect and allowing the post hoc presentation of the results pooled across the two groups.

### Conclusion

The present study has provided evidence that suggests treatment with the oral SIRT1 activator, SRT2104, may lead to an improvement in measures of arterial compliance in otherwise healthy cigarette smokers and people with type 2 diabetes. The exact mechanism of this improved arterial compliance and the effects of prolonged treatment with SRT2104 on vascular health remain to be elucidated. Given that aortic stiffness and endothelial function are key factors in predicting cardiovascular outcomes, identification of novel pharmacological means of improving these predictive parameters is important and highly relevant in populations with known cardiovascular risk factors.
