# Supplementary material for: Effects of the small molecule SIRT1 activator, SRT2104 on arterial stiffness in otherwise healthy cigarette smokers and subjects with type 2 diabetes mellitus
Source: Open Heart. 2016 May 17;3(1):e000402. doi: 10.1136/openhrt-2016-000402 (PMC4879341; doi:10.1136/openhrt-2016-000402)
Supplement: Supplementary table 1 — Parameters of arterial compliance for otherwise healthy cigarette smokers and subjects with type 2 diabetes mellitus [file openhrt-2016-000402supp_table1.pdf]

	Mean (SD)	Augmentation Index (%)		Corrected Augmentation Index (%)		Augmentation Pressure (mmHg)		Time to Wave Reflection (ms)		Pulse Wave Velocity (m/sec)	
		Baseline	Day 28/56	Baseline	Day 28/56	Baseline	Day 28/56	Baseline	Day 28/56	Baseline	Day 28/56
**Otherwise Healthy Smokers**	Placebo *(n=24)*	16.73 (16.91)	12.58 (18.09)	11.19 (15.35)	6.39 (17.60)	5.79 (5.46)	4.84 (7.11)	147.50 (15.45)	147.16 (12.79)	6.62 (1.44)	6.34 (1.35)
	SRT2104 *(n=24)*	11.30 (16.02)	9.73 (21.51)	5.64 (14.19)	3.68 (19.97)	4.89 (7.03)	3.69 (9.11)	148.48 (12.87)	149.60 (21.07)	6.15 (0.89)	6.26 (1.12)
**Subjects with type 2 diabetes**	Placebo *(n=14)*	21.34 (10.04)	18.35 (11.86)	22.28 (5.15)	15.77 (10.84)	9.97 (8.25)	8.81 (7.09)	144.69 (13.57)	147.52 (6.50)	9.59 (1.52)	9.05 (1.48)
	SRT2104 *(n=15)*	12.96 (12.36)	15.63 (11.81)	12.46 (7.91)	13.07 (10.45)	5.89 (5.91)	6.60 (5.99)	150.04 (6.19)	148.67 (8.90)	7.51 (1.55)	8.78 (2.01)

Supplementary table S1: Parameters of arterial compliance for otherwise healthy cigarette smokers and subjects with type 2 diabetes mellitus
